# Shoulder Presentation Occurring Thirteen Times in the Same Patient

**Published:** 1889-10

**Authors:** G. Eustache

**Affiliations:** Lille


[*Translation.*]

### SHOULDER PRESENTATION OCCURRING THIRTEEN TIMES IN THE SAME PATIENT.

BY DR. G. EUSTACHE, OF LILLE.

Shoulder presentations are comparatively rare. It is still more rare to see this presentation, which is abnormal and unphysiological, repeated a large number of times and even indefinitely, so to speak, in the same woman.

Observations of this kind are not very numerous in medical literature. The following are cited from an article by Polaillon which appeared in the *Annales de Gynecologie* of September, 1877.

Gery has reported a case in which the child presented by the arm in nine successive labors.

Walter attended a woman in five labors, in each of which the trunks presented. The fault was a twin labor and both twins presented by the shoulder.

Lechryse has seen the same phenomenon occuring three times in the same woman.

Tarnier (*Traité d'Accouchements*, *A. I.*) reports that Dangan has observed several cases in which the shoulder presented in the same woman in from five to nine successive labors, and says that he has observed the same thing at the Paris Maternity Hospital.

Naegele has observed it five times in six pregnancies. He quotes Meissner (*Traite d'Accouchements*, p. 592), who reports a case where a woman, after having borne her first child by a head presentation, had to be delivered eleven times in succession by version, on account of faulty presentation.

I have myself seen six women in whom I have had to practice version twice each for shoulder presentations. The majority of the attending physicians of the Maternite have seen cases of this kind, at least in those parts of the country where large families are the rule. But I think that there exist few or no observations comparable to the following in which a shoulder presentation has occurred in thirteen consecutive labors; and it is for that reason that I have seen fit to report it.

The patient Z. V. was married at the age of nineteen and had her first child in 1870. Delivery occurred spontaneously after a long and hard labor. The child presented by the head and lived. An almost complete rupture of the perineum occurred and a laceration of the left side of the cervix to the vaginal junction. These lacerations healed over after a fashion.

She became pregnant again a year later. At her labor the physician recognized a shoulder presentation and performed version. The child lived only a few hours.

In 1873 another pregnancy, shoulder presentation, version and a dead child. In 1875 and in 1876 the same.

In 1877 premature delivery at seven months of a dead child which presented by the arm.

1878 an abortion at three months.

In 1879 she entered my service at the Hospital Sainte Eugénie. She was four months pregnant. It was then that I recognized the existence of the laceration of perineum, and especially that of the cervix which, by reason of its extent, had produced a deformity of the organ. On account of her pregnancy I postponed operation until later. She left the hospital and was delivered prematurely at seven months, shoulder presentation, version, dead child.

In 1880 and in 1882 two more labors at term in which version was necessary. The children lived only a few hours.

In 1883 she presented herself at the Maternité Sainte-Anne. She was pregnant for the eleventh time and at term. The child presented by the right shoulder. During efforts at external version the membranes ruptured and the arm projected into the vagina. I then performed internal version and easily delivered a child which lived. On this occasion I sought for some malformation of the pelvis or of the uterus which might explain this extraordinary succession of shoulder presentations but found none.

Since that time the patient has become pregnant four times. Twice delivery took place prematurely at seven months, the third time at eight and a half months. Each time version had to be performed and the children were extracted dead.

Her fifteenth pregnancy dates from July, 1888. She came to the Maternité Sainte-Anne in January, 1889, for commencing labor at seven months. Quiet and injections of laudanum were prescribed. I recognized very clearly at this time that there was a presentation of the right shoulder and caused one of my pupils to practice external version. But on account of the small size and very great mobility of the fœtus on the one hand and the threatened premature labor on the other these efforts were not pushed to any great extent, and I advised the woman to return upon the reappearance of the pains.

Her pregnancy went on without accident and she did not return until the first of April. Labor had commenced and the os was dilated to the size of a quarter dollar. The uterus lay transversely and the child presented by the right shoulder. I immediately attempted to practice external version. I was able to push down the head, situated low down on the right side, as far as the inlet of the superior strait, but not to engage or fix it. On the contrary the effect of pressure was to depress the fœtus as a whole, and to push down still more the shoulder and the arm which projected into the bag of waters and became engaged in the cervix. In spite of repeated attempts at combined external version, I was unable to make the head descend. During these manœuvres, the os became completely dilated, and I decided upon internal version, which was performed with great ease by one of the internes.

I noticed particularly that the umbilical cord passed between the legs of the child and up along the back and finally encircled the right arm which explained the impossibility of external version.

The same cause led to a premature detachment of the placenta on account of which I delivered artificially as rapidly as possible.

I had no difficulty in exploring the cavity of the uterus in order to notice its conformation, and especially to ascertain if there existed any tendency to division of the uterus, (*uterus septus.*) But neither by the hand exploring the cavity of the uterus, nor by the hand applied to the abdomen over the fundus, was I able to detect any malformation of the uterine body.

The placental insertion was by the side of the opening of the left tube. The child died eight hours after birth.

The course of the puerperium was perfectly normal. The woman left the hospital on the eleventh of April.

Such is the history of this interesting case. The notable features of it may be summarized as follows:

A woman becomes pregnant for the first time and the child is born with a vertex presentation, the delivery being spontaneous. She subsequently becomes pregnant thirteen times, and in thirteen labors the child presents thirteen times by the shoulder, and thirteen times version has to be performed.

In such a case it cannot be said that the shoulder presentation is irregular, accidental, fortuitous, but rather that it is regular and normal. But it is in the explanation of such rare cases that in accidental or fortuitous cases one may offer in explanation causes which involve absence or derangement of the factors of accommodation, such as small size of the fœtus, dropsy of the amnion, twin pregnancy, deformity of the pelvis, abnormal insertion of the placenta, abnormal laxity of the uterine or abdominal walls, etc. But is difficult to admit the efficiency of causes purely contingent when transverse presentation occurs as a permanent condition.

Wiegand, Lachapelle and Hergott have stated that in these cases there exists a special rudimentary conformation of the uterus, a true malformation, by virtue of which the womb assumes the form of an ovoid whose greatest diameter, instead of being vertical, is horizontal, thus producing a shoulder presentation in which the fœtal and uterine axes, although parallel, are perpendicular to the pelvic axis. This theory is perfectly plausible and there is not an accoucheur who would refuse to admit such an etiology. But unfortunately it is only a theory and needs to be confirmed by facts.

In one instance such confirmation is furnished by Polaillon in in the case of a woman pregnant for the first time, in whom he found a shoulder presentation and insertion of the placenta upon the cervix. At the autopsy he found a heart-shaped uterus from arrest of development. As a result of this deformity, the transverse diameter of the womb was longer than the vertical diameter. The fœtus developed in such a space would lie transversely by virtue of the law of accommodation of the contained body to the containing cavity.

But I do not think that such a coincidence has been observed in any other case. In the one which I have reported, I can state that the most careful observation discovered nothing of the sort.

What then was the etiological factor in the case ? I see only one, namely, the relaxation of the uterine walls and of the fibro-muscular tissue which connects the uterus to the entrance of the pelvis, a relaxation which had its original cause in the extensive lateral laceration of the cervix during the first labor with vertex presentation.

To offer laceration of the cervix as an explanation of shoulder presentations is doubtless a new theory and perhaps a questionable one. I offer it simply for lack of a better. Be that as it may, if the woman were younger and if she were to ask my advice, I would not hesitate to recommend and perform trachelorrhaphy as a means of preventing further presentations of the trunk.—*Nouvelles Archives d' Obstetrique et de Gynecologie. May, 1889.*

